# Identification of Marine Neuroactive Molecules in Behaviour-Based Screens in the Larval Zebrafish

**DOI:** 10.3390/md12063307

**Published:** 2014-05-30

**Authors:** Si-Mei Long, Feng-Yin Liang, Qi Wu, Xi-Lin Lu, Xiao-Li Yao, Shi-Chang Li, Jing Li, Huanxing Su, Ji-Yan Pang, Zhong Pei

**Affiliations:** 1 Department of Neurology, National Key Clinical Department and Key Discipline of Neurology, Guangdong Key Laboratory for Diagnosis and Treatment of Major Neurological Diseases, The First Affiliated Hospital, Sun Yat-sen University, Guangzhou 510080, China; E-Mails: longsm@mail.sysu.edu.cn (S.-M.L.); liangfy@mail2.sysu.edu.cn (F.-Y.L.); wuqi@medmail.com.cn (Q.W.); goodxilin@163.com (X.-L.L.); liliyao71@163.com (X.-L.Y.); 2 School of Chemistry & Chemical Engineering, Sun Yat-sen University, Guangzhou 510275, China; E-Mails: lishich@mail2.sysu.edu.cn (S.-C.L.); zsulijing@163.com (J.L.); 3 State Key Laboratory of Quality Research in Chinese Medicine, Institute of Chinese Medical Sciences, University of Macau, Macao 999078, China; E-Mail: Huanxingsu@umac.mo

**Keywords:** behavior-based screen, zebrafish, PTZ, *c-fos*

## Abstract

High-throughput behavior-based screen in zebrafish is a powerful approach for the discovery of novel neuroactive small molecules for treatment of nervous system diseases such as epilepsy. To identify neuroactive small molecules, we first screened 36 compounds (**1**–**36**) derived from marine natural products xyloketals and marine isoprenyl phenyl ether obtained from the mangrove fungus. Compound **1** demonstrated the most potent inhibition on the locomotor activity in larval zebrafish. Compounds **37**–**42** were further synthesized and their potential anti-epilepsy action was then examined in a PTZ-induced epilepsy model in zebrafish. Compound **1** and compounds **39**, **40** and **41** could significantly attenuate PTZ-induced locomotor hyperactivity and elevation of *c-fos* mRNA in larval zebrafish. Compound **40** showed the most potent inhibitory action against PTZ-induced hyperactivity. The structure-activity analysis showed that the OH group at 12-position played a critical role and the substituents at the 13-position were well tolerated in the inhibitory activity of xyloketal derivatives. Thus, these derivatives may provide some novel drug candidates for the treatment of epilepsy.

## 1. Introduction

Disorders of the central nervous system (CNS) are very common and devastating. However, CNS diseases are usually poorly treated due to of the limited availability of selective neuroactive drugs. Thus, the development of novel neuroactive drugs is of high priority. It has been very difficult to discover novel neuroactive drugs in the past years [1]. Many current behavior-altering drugs were discovered by chance in the 1940s and 1950s. A major obstacle to the discovery of novel neuroactive drugs is the lack of available relevant model systems for screening large numbers of active compounds. Modeling the brain activity *in vitro* is problematic because of the complex networks of the brain. In addition, the screens in mice and rats are low-throughput due to the expense and ethical issues [2]. Recently, zebrafish has become a powerful model system for whole organism small molecule screening. Zebrafish are small, cheap to keep, fast to develop, and easy to breed. Similar to mammals, zebrafish larvae can display diverse behaviors including the optokinetic response [3], the optomotor response [4], prepulse inhibition [5] and sleep [6,7]. Combined with the video track system, several high-throughput behavior-based assays have been successfully applied to identify novel neuroactive small molecules in the zebrafish [8,9].

**Chart 1:**
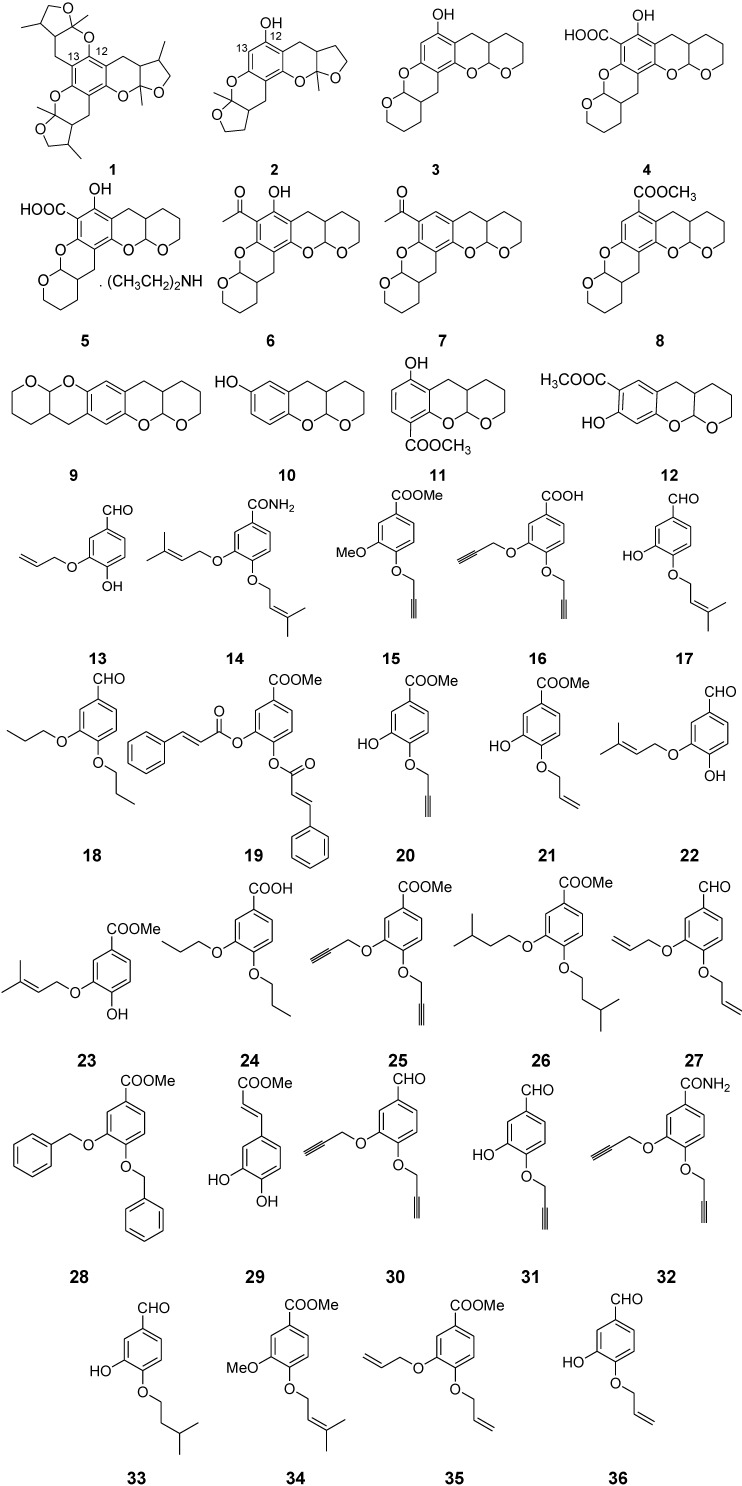
Structures selected for neuroactive screening.

**Chart 2:**
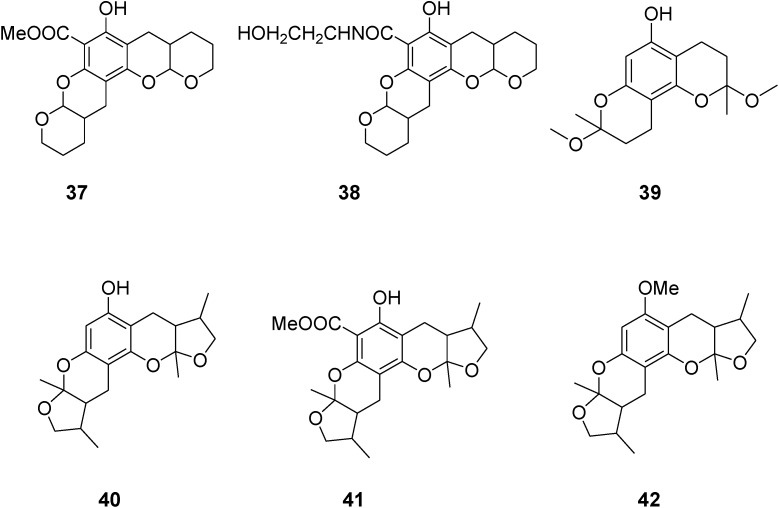
Modification of compound **1**.

The marine habitat is a rich resource for the discovery of new drugs because of its vast chemical and biological diversity. However, most marine-derived lead compounds are stereochemically complex or have low activity. Thus, the appropriate structural modifications of lead compounds are important to develop chemically simple and active drug candidates [10]. In this paper, we conducted a behavior-based screen for neuroactive small molecules on 12 benzopynan compounds derived from natural xyloketals from marine mangrove fungus (NO. 2508) [11] and 24 isoprenyl phenyl ether derivatives modified from marine isoprenyl phenyl ether from Mangrove fungus (NO. B60) [12] (Chart 1). We further modified compound **1** (Chart 2) to study structure-activity relationships and optimize the biological activity of compound **1** derived compounds. Finally, we explored the potential of compound **40** as a new antiepileptic candidate in pentylenetetrazol (PTZ)-induced epilepsy model in zebrafish.

## 2. Results and Discussion

### 2.1. Chemistry

**Scheme 1:**
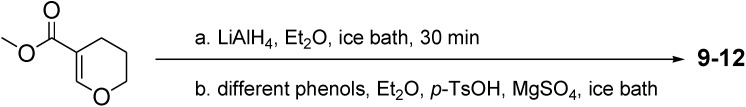
Synthesis of compounds **9**–**12**.

Forty-two analogues of the natural xyloketals and isoprenyl phenyl ether were obtained, and the general synthetic routes of compounds **1**–**42** have been described previously [10,12,13,14]. Compounds **9**–**12** were new compounds synthesized by reduction and electrophilic aromatic substitution reactions of 3,4-dihydro-2*H*-pyran-5-carboxylate with different phenols with 29%–52% yield (Scheme 1). The reduction product of 3,4-dihydro-2*H*-pyran-5-carboxylate was unstable and was immediately used for the subsequent experiment. The title compounds were isolated as their bis- and mono- adducts in different phenol proportions. Different substituted compounds of methyl 2,4-dihydroxybenzoate **11** and **12** could be obtained in one pot reaction and detected using TLC, followed by purifying, easily done by flash chromatography. However, the final products were obtained in low yields and further optimizations were required. Compounds **9**–**12** were fully characterized by HRMS and NMR.

### 2.2. Neuroactive Activity of 36 Compounds were Evaluated in Zebrafish Behavioral Assay

**Figure 1:**
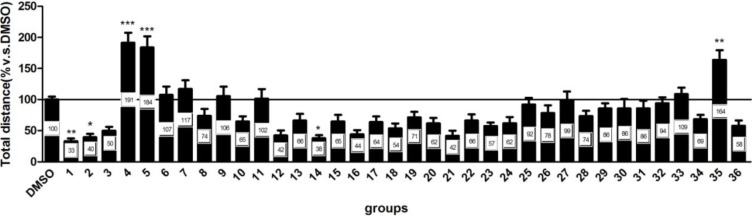
The larval zebrafish behavioral assay was performed on 120-hpf zebrafish dosed with compounds at 20 µM concentrations in DMSO. Each group had 24 replicates and three independent experiments were performed. The data of total distance are normalized as the percentage of control and representative of three independent experiments. Data was analyzed using One-way ANOVA followed by Post Hoc test (Bonferroni’s Multiple Comparison Test). * *p* < 0.05 *vs.* DMSO, ** *p* < 0.01 *vs.* DMSO, *** *p* < 0.001 *vs.* DMSO.

Zebrafish have neural networks similar to mammals and their locomotor activity is a measurable complex behavior. Similar to higher vertebrates, locomotor activity of zebrafish is also regulated by light. In zebrafish behavioral assays, the total swimming distance traveled is often measured to determine the changes in the locomotor activity. Therefore, we examine the effect of different compounds on the total distance during the initial screen. All marine-derived compounds (**1**–**36**) were dissolved in DMSO and diluted with E3 buffer (5 mM NaCl, 0.17 mM KCL, 0.33 mM CaCl_2_·2H_2_O, 0.33 mM MgSO_4_) to a final concentration of 20 μM. The results (Figure 1) showed that three compounds including compounds **1**, **2** and **14** significantly inhibited locomotor activity (*p* < 0.01 *vs.* DMSO) to 33%, 40% and 38%, respectively. Meanwhile, several compounds exhibited a hyperactive effect on locomotor activity (*p* < 0.01 *vs.* DMSO). For example, compounds **4**, **5** and **35** could significantly increase locomotor activity by 91%, 84% and 64%, respectively.

### 2.3. Neuroactive Compounds Exhibited Different Behavioral Patterns

**Figure 2:**
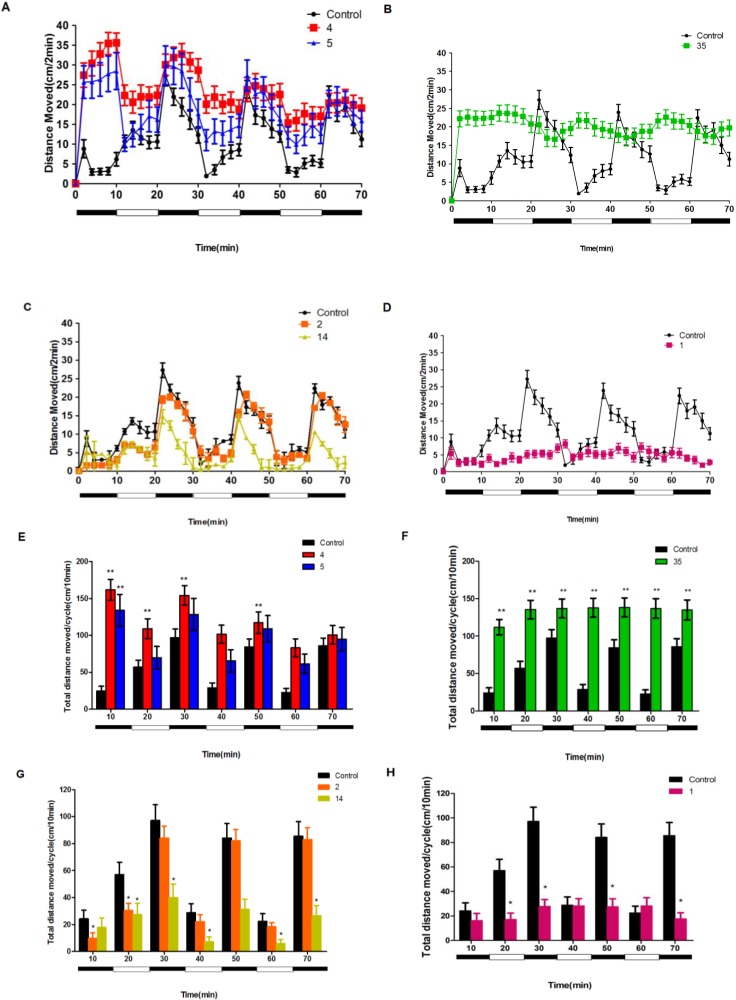
The behavioral assay of neuroactive compounds. The larvae were placed into the compound solutions (final concentration is 20 µM) and recording began 20 min later in alternating periods of darkness and light for a total duration of 70 min. (**A**–**D**) Total activity (distance moved, cm) in two-minute intervals for total duration of 70 min. (**E**–**H**) Total activity (distance moved, cm) in each 10-minute light and dark period. Values are reported as mean (*n* = 21–24 larvae/concentration/plate, for 2 plates) ±SEM. * *p* < 0.05 *vs.* DMSO, ** *p* < 0.01 *vs.* DMSO.

The behavioral assay used here has been well-characterized. During this assay, zebrafish typically exhibited robust but transient behavioral activity in response to sudden transitions from light to dark [15]. In the present study, we used a modified version of this test consisting of a single transition from light to dark. The basal swimming activity was recorded during 10 min with lights on. Immediately following the basal activity recording, the lights were suddenly turned off for 10 min. Consistent with previous reports, the control animals displayed a normal pattern of locomotor activity, *i.e.*, the activity of zebrafish decreased when the visible light was on (light) whereas the activity of zebrafish rapidly and markedly increased when the light was off (dark). We further analyzed these six neuroactive (compounds **1**, **2**, **4**, **5**, **14**, **35**) compounds obtained from the initial screen. We found that neuroactive compounds exhibited distinct patterns and some neuroactive compounds altered the orderly normal activity pattern (Figure 2). Based on the behavior assay, marine-derived compounds tested could be simply categorized as two major types: hyperactive and hypoactive compounds. The behaviors also varied among different compounds within the same types. For example, among these hyperactive compounds, compounds **4** and **5** showed hyperactive activities which were more prevalent in darkness than in light (Figure 2A,E) whereas compound **35** induced a constant hyperactivity all the time regardless of dark or light conditions (Figure 2B,F). Among hypoactive compounds, compound **2** decreased activities during the first dark and light cycle and activities were then returned to normal in the subsequent cycles whereas compound **14** decreased activity during all the periods (Figure 2C,G). In addition, animals treated with compound **1** decreased activity in every dark and light period (Figure 2D,H).

### 2.4. Compounds **1** and **37**–**41** Suppressed Locomotor Activity in Larval Zebrafish

**Figure 3:**
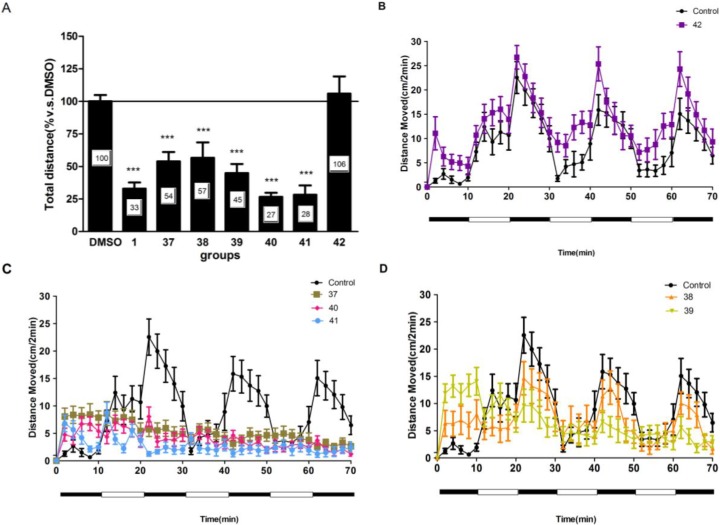
Compounds **1** and **37**–**41** suppressed larval zebrafish locomotor activity. (**A**) The behavior assay was performed with compounds **37**–**42**. The relative distance of DMSO group was set as control and other groups were shown relative to DMSO group. (**B**–**D**) Different activity patterns of compound **1** and compounds **37**–**42**. * *p* < 0.05 *vs.* DMSO, *** *p* < 0.001 *vs.* DMSO.

To further study the structure-activity relationships and optimize biological activity of compound **1**, three compounds (**40**–**42**) derived from **1** and three more compounds(**37**–**39**) were synthesized (Scheme 1). The results (Figure 3A) showed that the majority of compounds inhibited the locomotor activity (total distance) at the concentration of 20 μM (*p* < 0.05 *vs.* DMSO). Among them, compounds **37**–**41** could significantly reduce total distance by up to 57%. Moreover, compounds **40** and **41** displayed more potent inhibition compared to the lead compound **1** (reduced activity level to 27% and 28%, respectively). Six derivatives displayed different patterns of locomotor activity (Figure 3B–D). Compound **42** displayed a reverse effect with a higher activity during all of the dark and light cycles. Similar to compound **1**, compounds **37**, **40** and **41** induced a constant inhibition on locomotor activity throughout every dark and light period. Furthermore, compounds **40** and **41** displayed much lower activity compare to compound **1**. Although compounds **38** and **39** suppressed the locomotor activity, they did not disturb the normal orderly pattern of activity. Altogether, we found that compound **40** showed the most potent inhibitory action on locomotor activity.

Structure–activity investigation was conducted to identify the active components within xyloketal derivatives. We found that xyloketal derivatives possessing the benzopyrano furan skeleton exhibited more potent inhibitory action compared to that possessing benzopyrano pyran. However, methyl ether derivative **42** did not show any suppression of activity compared to controls. Thus, an unsubstituted hydroxyl group or easily cleaved hydroxyl prodrug, such as a phenolic ester at 12-position of the benzopyran scaffold, may be important for the inhibitory action. Furthermore, the substituents at the 13-position may be tolerated but not necessary for the inhibitory activity. However, compound **1** does not possess an OH group at this position perhaps hinting at different binding modes between **1** and **40**/**41**.

### 2.5. The Potential of Selected Compounds for New Antiepileptic Drug Development in PTZ-Induced Seizure Model

Epilepsy is one of the most common CNS disorders and affects about 50 million people worldwide [16]. A variety of pharmacological and genetic models of epilepsy have been developed to study seizure mechanisms and to identify new anti-epileptic drugs. PTZ is a noncompetitive antagonist of the GABA receptor complex and its epileptogenic properties have been widely used for anti-epileptic drug discovery. Similarly, PTZ can induce measurable seizure-related behaviors in zebrafish. These behaviors can be reversed by known anti-epileptic drugs. Thus, zebrafish are emerging as a useful model system for anti-epileptic drug discovery.

**Figure 4:**
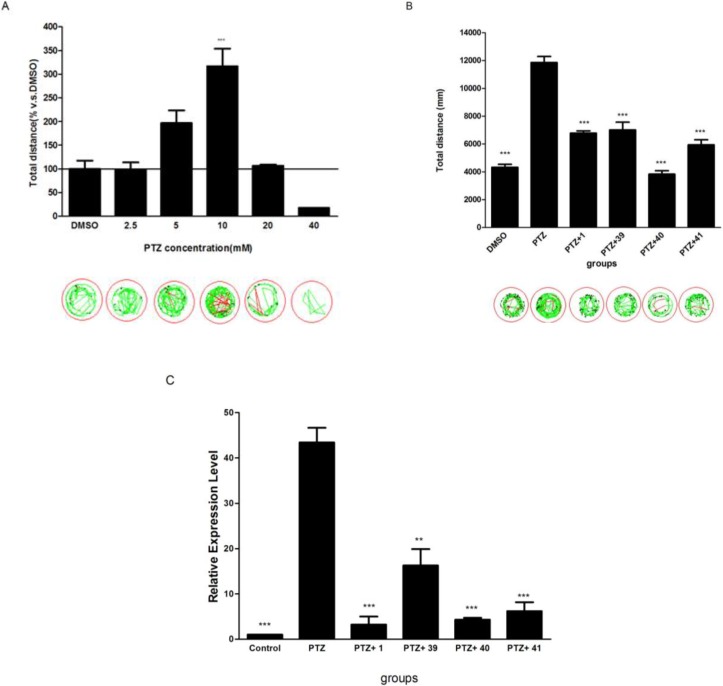
The effect of compounds **1** and **39**–**41** on PTZ-induced hyperactivity in zebrafish. (**A**) The effect of PTZ on larval activity was initially tested across a broad concentration range (0–40 mM). *** *p* < 0.001 *vs.* DMSO. (**B**) The total distances in larval zebrafish exposure to 10 mM PTZ with or without compound **1** and compounds **39**–**41**. *** *p* < 0.001 *vs.* PTZ. (**C**) The expression levels of *c-fos* mRNA in larval zebrafish exposure to PTZ (10 mM for 60 min) with or without tested compounds (** *p* < 0.01 *vs.* PTZ, ***, *p* < 0.0001).

The potential of compounds **1** and **39**–**41** as anti-epileptic drugs was further explored in a PTZ-induced epilepsy model in zebrafish. Consistent with previous reports [17], PTZ at 10 mM induced a robust locomotor activity (hyperactivity) in zebrafish larvae (Figure 4A). In contrast, all three compounds (**39**–**41**) demonstrated significant inhibitory action (hypoactivity) in this PTZ model. Among compounds tested, compound **40** showed the most potent inhibitory activity (Figure 4B).

PTZ induced changes not only in activity but also in gene expression. The expression levels of IEGs are well correlated with seizure activity. The expression of *c-fos* has been used as an indicator of neuronal activity in zebrafish which is often measured by using the electroencephalography (EEG) [18], To investigate whether compounds **1** and **39**–**41** affects PTZ-induced expression of the gene *c-fos*, quantitative real time PCR of *c-fos* was performed on larval zebrafish (Figure 4C). The results indicated that the PTZ treatment (10 mM for 60 min) induced about 43-fold expression of *c-fos* mRNA in five dpf larval zebrafish whereas compounds **1** and **39**–**41** significantly attenuated the PTZ-induced increase in *c-fos* expression. The inhibitory action on *c-fos* expression by compounds tested corresponded to their suppression of PTZ-induced movement in the larval zebrafish. Consistent with the behavior assay, compound **40** exhibited one of the most potent inhibitory actions. Given that PTZ induces *c-fos* expression exclusively in central nervous system in zebrafish, our data also suggests that the compounds are able to cross the blood brain barrier [19].

### 2.6. The Dose-Response Study of Compound **40** in PTZ Model

**Figure 5:**
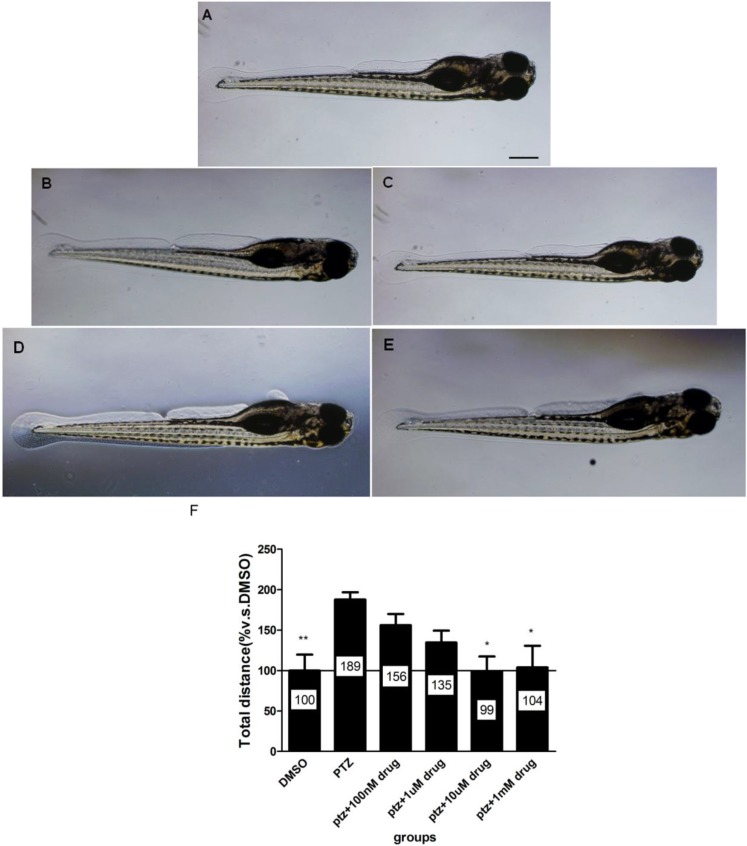
The effect of compound **40** on PTZ-induced morphological changes. (**A**) The morphology of larval zebrafish at five dpf. (**B**–**E**) The morphology of five dpf larval zebrafish exposed to compound **40** at different concentrations (100 nM, 1, 10, 100 µm and 1 mM). Scale bar represents 100 µm. (**F**) The total distances in larval zebrafish exposure to PTZ with or without different concentrations of compound **40**. * *p* < 0.05 *vs.* PTZ, ** *p* < 0.01 *vs.* PTZ.

We then conducted a dose-response study on compound **40**, the most potent inhibitory compound tested, to explore its efficacy and toxicity. We found that compound **40** at doses between 10 µm and 1 mM significantly reduced PTZ-induced hyperactivity in larval zebrafish (Figure 5F). To investigate whether compound **40** causes potential toxicity to zebrafish, we examined the morphology of compound **40**-treated larval zebrafish. The results (Figure 5A–E) showed normal morphology in larval zebrafish receiving different doses of **40**, indicating that compound **40** alone at doses between 1 µm and 1 mM did not cause toxic effects on larval zebrafish. Images of larval zebrafish were taken using an OLYMPUS IX71 inverted microscope at 4× magnification.

Calcium has been implicated in the pathophysiology of seizure. It has been reported that calcium influx mediates seizure induction whereas blockage of calcium channels reduces epileptic activity [20,21,22]. Xyloketal derivatives have multiple-properties and their inhibitory action on L-calcium channel activity has been reported [23]. Therefore, xyloketal derivatives may exert their anti-convulsive effect by inhibiting the activity of calcium channels. In addition to the direct anti-convulsive action, calcium channel antagonists can potentiate the anticonvulsant action of other anti-epileptics such as diazepam. Xyloketal derivatives deserve further investigation as a potential anticonvulsant drug candidate.

## 3. Experimental Section

### 3.1. Chemistry

All commercial available reagents and solvents were used directly without further purification unless otherwise stated. ^1^H and ^13^C NMR data were recorded on a 300 MB NMR spectrometer operating at 400 MHz and 100 MHz for ^1^H and ^13^C, respectively. Tetramethylsilane (TMS) as internal standard and all chemical shifts are in ppm (δ). Mass spectra were detected on DSQ (Low Resolution Mass Spectrometer) and MAT95XP (High Resolution Mass Spectrometer).

### 3.2. General Procedure of Synthesizing Compounds **9**–**12**; Take **9** for Example

#### 3.2.1. Compound **9**

To a suspension of lithium aluminum hydride (0.76 g, 20 mmol) in ether (30 mL) with an ice water bath was added a solution of 3,4-dihydro-2*H*-pyran-5-carboxylate (1.88 g, 13.2 mmol, 3.2 molar equivalent to the subsequent reagent hydroquinone) in ether (20 mL) dropwise. The resultant mixture was allowed to warm to room temperature and stirred for additional 1 hour. Then, aqueous solution of 2 M sodium hydroxide (1.2 g) was added at 0 °C. The resultant mixture was filtered, washed with ether (3 × 50 mL), and the combined filtrates were concentrated in vacuo below 10 °C to afford the reduction product as a colorless liquid. This material was immediately diluted with ether (20 mL) and used for the subsequent experiment without storage because of its instability.

To a suspension of hydroquinone (0.52 g, 4.1 mmol) and anhydrous magnesium sulfate (2 g) in 40 mL ether at 0 °C was added the prepared above ether solution as soon as possible. After stirring for 1 min, *p*-toluene sulfonic acid (0.1 g) was added. The resultant mixture was allowed to warm to room temperature and stirred for 30 min. The reaction mixture was then filtered and the filter-cake was washed with ether (3× 50 mL). The combined filtrates were washed with water (100 mL) and saturated brine (100 mL) followed by drying over anhydrous magnesium sulfate and concentrated in vacuo. Purification by flash chromatography using ether: dichloromethane (2:25) afforded 0.71 g title compound **9** as a colorless crystal in 50% yield. ^1^H NMR (400 MHz, CDCl_3_) δ 6.68 (s, 1H), 6.52 (s, 1H), 5.28–5.14 (m, 2H), 4.06–3.93 (m, 2H), 3.72–3.63 (m, 2H), 2.93–2.39 (m, 4H), 2.25–2.05 (m, 2H), 1.71–1.59 (m, 8H). EI-MS *m*/*z* 302 (M). EI-HR-MS *m*/*z* Found: 302.1510, Calcd. for C_18_H_22_O_4_: 302.1513.

#### 3.2.2. Compound **10**

The title compound **10** was obtained 0.48 g from hydroquinone as a colorless crystal in 52% yield. ^1^H NMR (400 MHz, CDCl_3_) δ 6.69 (d, *J* = 8.7 Hz, 1H), 6.58 (dd, *J* = 8.7, 3.0 Hz, 1H), 6.49 (d, *J* = 2.9 Hz, 1H), 6.13 (s, 1H), 5.25 (d, *J* = 2.6 Hz, 1H), 4.05–3.96 (m, 1H), 3.73–3.66 (m, 1H), 2.81 (dd, *J* = 16.7, 6.1 Hz, 1H), 2.57 (dd, *J* = 16.7, 5.3 Hz, 1H), 2.17–2.06 (m, 1H), 1.69–1.59 (m, 4H). ^13^C NMR (100 MHz, CDCl_3_) δ 149.73, 146.07, 120.57, 116.82, 115.35, 114.46, 96.45, 62.76, 31.51, 28.50, 24.11, 23.12. EI-MS *m*/*z* 206 (M). EI-HR-MS *m*/*z* Found: 206.0940, Calcd. for C_12_H_14_O_3_: 206.0937.

#### 3.2.3. Compound **11**

The title compound was obtained 0.18 g from methyl 2,4-dihydroxybenzoate as a colorless crystal in 29% yield. ^1^H NMR (400 MHz, CDCl_3_) δ 11.18 (s, 1H), 7.62 (d, *J* = 8.9 Hz, 1H), 6.43 (d, *J* = 8.9 Hz, 1H), 5.34 (d, *J* = 2.5 Hz, 1H), 4.06–3.96 (m, 1H), 3.90 (s, 3H), 3.79–3.68 (m, 1H), 2.85–2.61 (m, 2H), 2.29–2.12 (m, 1H), 1.76–1.58 (m, 4H). ^13^C NMR (100 MHz, CDCl_3_) δ 170.77, 161.21, 158.86, 128.67, 108.42, 107.88, 105.09, 96.96, 62.57, 51.87, 30.92, 24.09, 23.63, 23.02. EI-MS *m*/*z* 264 (M). EI-HR-MS *m*/*z* Found: 264.0998, Calcd. for C_14_H_16_O_5_: 264.0992.

#### 3.2.4. Compound **12**

The title compound **12** was obtained 0.21 g from methyl 2,4-dihydroxybenzoate as a colorless crystal in 34% yield. ^1^H NMR (400 MHz, CDCl_3_) δ 10.61 (s, 1H), 7.52 (s, 1H), 6.45 (s, 1H), 5.38 (s, 1H), 4.04–3.93 (m, 1H), 3.90 (s, 3H), 3.79–3.63 (m, 1H), 2.97–2.74 (m, 1H), 2.65–2.49 (m, 1H), 2.27–2.07 (m, 1H), 1.73–1.53 (m, 4H). ^13^C NMR (100 MHz, CDCl_3_) δ 170.16, 161.81, 159.56, 130.88, 111.86, 106.15, 103.82, 97.13, 62.22, 51.84, 31.77, 28.41, 23.63, 23.57. EI-MS *m*/*z* 264 (M).

### 3.3. Zebrafish Behavior-Based Activity Screen

#### 3.3.1. Zebrafish Maintenance

Zebrafish (Danio rerio) were maintained according to standard animal care protocols [24]. AB strain zebrafish were bred to yield embryos. Embryos and larvae were maintained on a re-circulating Tecniplast aquatic system at 28 ± 1 °C and between pH 7.0 and 7.5 on a 14/10 h light/dark (L/D) cycle. Embryos were collected from multiple AB/Tubingen breeding. Embryos were collected, rinsed in E3 buffer (5 mM NaCl, 0.17 mM KCl, 0.33 mM CaCl_2_·2H_2_O, 0.33 mM MgSO_4_), washed three times, transferred to Petri dishes and incubated at 28 °C until 4 dpf. They were washed twice every day to remove dead or unfertilized embryos.

#### 3.3.2. Zebrafish Behavioral Assay

AB strain zebrafish were bred to yield embryos. At 4 dpf, larval zebrafish were transferred to 96-well microplates and acclimated at 28 °C overnight. Twenty-four h later, compounds were added and incubated with zebrafish for 30 min at 28 °C. The 96-well microplates were then put into the zebrafish tracking box (Viewpoint Life Sciences Inc., Montreal, Canada) and the activity of zebrafish was monitored using automated video-tracking (the Viewpoint video tracking system and software).

After zebrafish acclimated at the tracking box for 20 min, their behaviors were recorded in infrared light (which will hereafter be referred to as “dark”), and the infrared light remained on throughout the recording session. In the behavior assay, the visible light (or “light”) was switched on for 10 min and then switched off for 10 min (dark period). This light/dark transition was continued for a total duration of 70 min. Each drug challenge was conducted on at least two separate plates with *n* = 12 larvae/plate in the compounds challenges, and 24 larvae/plate in the PTZ challenge (for controls, *n* = 12 larvae/plate in the compounds challenge and 24 larvae/plate in the PTZ challenge). All experiments consisted of 20 min of acclimation in the dark followed by seven 10 min-cycles containing a darkness and light phase (90 min total). To capture the different types of activity, a threshold was set at 25 mm/s in order to separate the fast/darting activity and 4 mm/s to separate the slow activity.

#### 3.3.3. Morphology Assay

Briefly, 5 dpf zebrafish with or without compounds were anaesthetized by treatment with 0.4% tricaine and mounted onto glass slide. The morphology was visualized at 4× magnification under an OLYMPUS IX71 inverted microscope.

#### 3.3.4. Quantitative RT Real Time PCR

Total RNA from zebrafish was extracted using E.Z.N.A. total RNA extraction kit (OMEGA biotek, Inc., Norcross, GA, USA). Intact RNA was checked by running a 1.0% agarose/formaldehyde gel and quantified spectrometrically (Beckman Coulter DU 800) before proceeding to subsequent steps. Five hundred ng of total RNA was reverse-transcribed using PrimeScript™ RT Master Mix (Perfect Real Time) Kit (Takara Inc., Otsu, Japan) according to manufacturer’s instructions. Real-Time PCR was performed on an Opticon MONITORTM Software (MJ Research Inc., Quebec, Canada) using SYBR^®^ Premix Ex Taq™ II (Tli RNaseH Plus) (Takara Inc.). Expression levels for each target gene were calculated by the 2~DDCT method [25]. All analyses were performed in triplicates. Primers used for the RT real-time PCR are as follow:

*c-fos* Forward primer: 5′-GCTCCATCTCAGTCCCAGAG-3′
*c-fos* Reverse primer: 5′-AGAGTGGGCTCCAGATCAGA-3′
*β-actin* Forward primer: 5′-CCGTTGCCCCGAGGCTCTCT-3′
*β-actin* Reverse primer: 5′-CGCATCCTGAGTCAATGCGCCA-3′

#### 3.3.5. Data Analysis

Dead larvae or larvae with physical abnormalities were not included in any data analyses or figures. Any observed death or abnormalities were not due to drug exposures, and the total amount of dead and abnormal animals on each plate represented less than 5% of the total population. Data were analyzed using Graph Pad Prism software. Replicate experiments were run on two separate days (*n* = 12/day) for both the therapeutic dilution series test and the PTZ dilution series test, with a carrier control group in each plate. The data for each drug were first assessed using a repeated measures analysis of variance (ANOVA) with time and dose as the independent variables and locomotor activity (distance moved/time) as the dependent variable. All data are presented as mean ± standard error of the mean (SEM).

## 4. Conclusions

In the present study, we have evaluated the neuroactive activity of 36 natural compounds and six designed novel derivatives in zebrafish model. Compound **1** and compounds **39**, **40** and **41** could significantly attenuate PTZ-induced locomotor hyperactivity and elevation of *c-fos* mRNA in larval zebrafish. Compound **40** showed the most potent inhibitory action against PTZ-induced changes. The structure-activity analysis showed that the OH group at the 12-position had a critical role and the substituents at the 13-position had a favorable role in the inhibitory activity of xyloketal derivatives. Thus, these derivatives may provide some novel drug candidates for the treatment of epilepsy.

## Abbreviations

HT: high-throughput
PTZ: pentylenetetrazol
GABA: γ-Aminobutyric acid
5-HT: 5-hydroxy-tryptamine
NMDA: *N*-Methyl-d-aspartic acid
CBZ: carbamazepine
DMSO: dimethyl sulfoxide
dpf: days post-fertilization
hpf: hours post fertilization
CNS: central nervous system
IEGs: immediate early genes
EEG: electroencephalography

## References

[1] Pangalos M.N., Schechter L.E., Hurko O. (2007). Drug development for CNS disorders: Strategies for balancing risk and reducing attrition. Nat. Rev. Drug Discov..

[2] Kokel D., Peterson R.T. (2008). Chemobehavioural phenomics and behaviour-based psychiatric drug discovery in the zebrafish. Brief. Funct. Genomics Proteomics.

[3] Brockerhoff S.E., Hurley J.B., Janssen-Bienhold U., Neuhauss S.C., Driever W., Dowling J.E. (1995). A behavioral screen for isolating zebrafish mutants with visual system defects. Proc. Natl. Acad. Sci. USA.

[4] Neuhauss S.C., Biehlmaier O., Seeliger M.W., Das T., Kohler K., Harris W.A., Baier H. (1999). Genetic disorders of vision revealed by a behavioral screen of 400 essential loci in zebrafish. J. Neurosci..

[5] Burgess H.A., Granato M. (2007). Sensorimotor gating in larval zebrafish. J. Neurosci..

[6] Prober D.A., Rihel J., Onah A.A., Sung R.J., Schier A.F. (2006). Hypocretin/orexin overexpression induces an insomnia-like phenotype in zebrafish. J. Neurosci..

[7] Zhdanova I.V., Wang S.Y., Leclair O.U., Danilova N.P. (2001). Melatonin promotes sleep-like state in zebrafish. Brain Res..

[8] Kokel D., Bryan J., Laggner C., White R., Cheung C.Y., Mateus R., Healey D., Kim S., Werdich A.A., Haggarty S.J. (2010). Rapid behavior-based identification of neuroactive small molecules in the zebrafish. Nat. Chem. Biol..

[9] Rihel J., Prober D.A., Arvanites A., Lam K., Zimmerman S., Jang S., Haggarty S.J., Kokel D., Rubin L.L., Peterson R.T. (2010). Zebrafish behavioral profiling links drugs to biological targets and rest/wake regulation. Science.

[10] Li S., Shen C., Guo W., Zhang X., Liu S., Liang F., Xu Z., Pei Z., Song H., Qiu L., Lin Y., Pang J. (2013). Synthesis and neuroprotective action of xyloketal derivatives in Parkinson’s disease models. Mar. Drugs.

[11] Liu R., Zhou Z.Y., Jiang M.Y., Wang F., Liu J.K. (2010). A new isoprenyl phenyl ether riboside from the culture of basidiomycete Laccaria amethystea. J. Asian Nat. Prod. Res..

[12] Li J., Zhang D., Zhu X., He Z., Liu S., Li M., Pang J., Lin Y. (2011). Studies on synthesis and structure-activity relationship (SAR) of derivatives of a new natural product from marine fungi as inhibitors of influenza virus neuraminidase. Mar. Drugs.

[13] Pettigrew J.D., Wilson P.D. (2006). Synthesis of xyloketal A, B, C, D, and G analogues. J. Organ. Chem..

[14] Xu Z., Li Y., Xiang Q., Pei Z., Liu X., Lu B., Chen L., Wang G., Pang J., Lin Y. (2010). Design and synthesis of novel xyloketal derivatives and their vasorelaxing activities in rat thoracic aorta and angiogenic activities in zebrafish angiogenesis screen. J. Med. Chem..

[15] Guo S. (2004). Linking genes to brain, behavior and neurological diseases: What can we learn from zebrafish?. Genes Brain Behav..

[16] (2012). Epilepsy Fact Sheet, World Health Organization. http://www.who.int/mediacentre/factsheets/fs999/en/.

[17] Mussulini B.H., Leite C.E., Zenki K.C., Moro L., Baggio S., Rico E.P., Rosemberg D.B., Dias R.D., Souza T.M., Calcagnotto M.E. (2013). Seizures induced by pentylenetetrazole in the adult zebrafish: a detailed behavioral characterization. PLoS One.

[18] Scallet A.C., Kowalke P.K., Rountree R.L., Thorn B.T., Binienda Z.K. (2004). Electroencephalographic, behavioral, and c-*fos* responses to acute domoic acidexposure. Neurotoxicol. Teratol..

[19] Fleming A., Diekmann H., Goldsmith P. (2013). Functional characterisation of the maturation of the blood-brain barrier in larval zebrafish. PLoS One.

[20] Ghasemi M., Shafaroodi H., Nazarbeiki S., Meskar H., Heydarpour P., Ghasemi A., Talab S.S., Ziai P., Bahremand A., Dehpour A.R. (2010). Voltage-dependent calcium channel and NMDA receptor antagonists augment anticonvulsant effects of lithium chloride on pentylenetetrazole-induced clonic seizures in mice. Epilepsy Behav..

[21] Meyer F.B., Anderson R.E., Sundt T.M., Yaksh T.L., Sharbrough F.W. (1987). Suppression of pentylenetetrazole seizures by oral administration of a dihydropyridine Ca^2+^ antagonist. Epilepsia.

[22] Moron M.A., Stevens C.W., Yaksh T.L. (1990). The antiseizure activity of dihydropyridine calcium channel antagonists in the conscious rat. J. Pharmacol. Exp. Ther..

[23] Wu X.Y., Liu X.H., Lin Y.C., Luo J.H., She Z.G., Jin L.H., Chan W.L., Antus S., Kurtan T., Elsässer B. (2005). Xyloketal F: A strong l-calcium channel blocker from the mangrove fungus *xylaria* sp. (#2508) from the South China Sea Coast. Eur. J. Org. Chem..

[24] Westerfield M. (1995). The Zebrafish Book: A Guide for Laboratory Use of Zebrafish (Danio rerio).

[25] Livak K.J., Schmittgen T.D. (2001). Analysis of relative gene expression data using real-time quantitative PCR and the 2(−Delta Delta C(T)) method. Methods.

